# Egg and Dietary Cholesterol Consumption and Mortality Among Hypertensive Patients: Results From a Population-Based Nationwide Study

**DOI:** 10.3389/fnut.2021.739533

**Published:** 2021-10-29

**Authors:** Fei Wu, Pan Zhuang, Yiju Zhang, Chuchu Zhan, Yu Zhang, Jingjing Jiao

**Affiliations:** ^1^Department of Nutrition, School of Public Health, Department of Clinical Nutrition of Affiliated Second Hospital, Zhejiang University School of Medicine, Hangzhou, China; ^2^National Engineering Laboratory of Intelligent Food Technology and Equipment, Zhejiang Key Laboratory for Agro-Food Processing, College of Biosystems Engineering and Food Science, Zhejiang University, Hangzhou, China

**Keywords:** eggs, dietary cholesterol, mortality, hypertensive patients, China health and nutrition survey (CHNS)

## Abstract

**Background:** Hypertensive patients are sensitive to the amount of dietary cholesterol intake, especially cholesterol from the whole eggs. Whether whole egg and dietary cholesterol consumption are suitable for hypertensive patients is still controversial.

**Aim:** The objective of the study was to examine the associations of intake of eggs as well as the dietary cholesterol with total mortality in a Chinese nationwide cohort.

**Methods:** We utilized data from the China Health and Nutrition Survey (CHNS) from the year of 1991 to 2015. Cumulative averages of egg and cholesterol intake were calculated to represent the consumption of the long-term diet of the participants in each available round of the survey. Cox regression models were employed to estimate the effects of eggs and dietary cholesterol from the different sources on mortality among hypertensive patients.

**Results:** A total of 8,095 participants were included in the final analysis and followed up for a mean of 11.4 years. Finally, 927 cases of death were detected. After adjustment for the multivariate factors, consuming more than seven eggs per week was related to 29% lower mortality among the hypertensive patients compared with the consumers with not more than two eggs per week [hazard ratio (HR): 0.71; 95% CI: 0.59–0.85; *P* < 0.001]. Similarly, the egg-sourced cholesterol intake was inversely associated with mortality (*P* = 0.002) whereas intake of the dietary cholesterol from the non-egg sources was significantly related to the higher mortality (*P* < 0.001). However, total cholesterol intake was not related to mortality among hypertensive patients. Substituting eggs for an equivalent amount of non-egg-sourced protein-abundant foods was also associated with lower mortality.

**Conclusion:** Higher consumption of eggs and egg-sourced dietary cholesterol was associated with lower mortality among the enrolled Chinese hypertensive patients but non-egg-sourced cholesterol intake was related to higher mortality. Therefore, our findings do not support the view that hypertensive patients should avoid whole egg consumption for the purpose of restricting dietary cholesterol intake.

## Introduction

The number of hypertensive patients is increasing and is estimated to exceed 1.6 billion by 2025 (1). Hypertensive patients have a higher risk of cardiovascular disease (CVD), which is a leading cause of death worldwide (2). Given that a slight increase in blood pressure could raise it above the levels considered safe in hypertensive patients, they may be more sensitive to the diet than normal individuals. Therefore, more attention should be paid to a healthy diet that could improve the health status of hypertensive patients. Due to the high cholesterol content (approximately 186 mg cholesterol per egg in the yolk) (3), it is highly debated whether consuming eggs is beneficial for health. There is a common phenomenon in China that elder citizens with diseases [e.g., CVD, coronary heart disease (CHD)] avoid yolk even whole egg consumption whereas fitness people prefer intaking more than 2 eggs/d without yolks. Meanwhile, the eggs are rich in various and necessary nutrients (e.g., protein, vitamins, minerals, lecithins, and carotenoids) (4, 5) beneficial to human health. Established studies were too inconsistent and lacking to deal with the major problem of whether people should consume the egg yolks or not, especially for hypertensive patients. A cohort study found that the egg intake was associated with higher total and cancer mortality [HR *via* comparing consumers with higher intake (≥2 eggs/d) with the reference category (1 egg/d) (95% CI): 2.05 (1.20–3.52) for total mortality and 3.20 (1.51–6.76) for the cancer mortality], whereas was not associated with CVD mortality in women in Japan (6). Results from the Physician's Health Study also indicated that frequent egg consumption (≥7 eggs/wk) was associated with the higher mortality compared with the consumers with <1, 1, 2–4, or 5–6 egg(s)/wk and a dose-response relationship could be observed (*P* < 0.001) (7). Although a review of the observational studies concluded no significant associations of egg consumption with some health outcomes, such as cancer, CVD, and metabolic diseases, they observed a protective relationship between egg consumption and stroke risk (8). A recent meta-analysis also showed an inverse association of up to 6 eggs/wk consumption with CVD incidence and/or mortality (9). Among the US population, they observed a lower risk of CVD for 4 eggs/wk [relative risk (RR) (95%): 0.94 (0.89–0.99), *I*^2^ (%) = 20]. However, a pooled cohort study of six prospective US cohorts showed higher consumption of dietary cholesterol or eggs was vastly associated with a higher risk of incident CVD and total mortality among the US adults (10).

Therefore, the association between egg consumption and health outcome is still unclear. Moreover, no studies explored the association of egg or dietary cholesterol consumption with the mortality among hypertensive patients in Chinese. Therefore, to better provide dietary cholesterol guidelines especially for the hypertensive patients, we assessed the relationship between the consumption of eggs and cholesterol from the different dietary sources and mortality among Chinese with hypertension in CHNS.

## Materials and Methods

### Study Population

The details of CHNS had been described elsewhere (11, 12). Briefly, the CHNS is a household-based and nationwide study initiated in 1989 and followed up every 2–3 years subsequently to assess how the society and economy transformation affect the health-related outcomes and nutrition status in China. The survey used a multistage random-cluster sampling process to draw samples from the nine provinces, plus three autonomous cities that were added in 2011. All the members in the selected households were surveyed by well-trained interviewers. Until now, 10 waves (1989, 1991, 1993, 1997, 2000, 2004, 2006, 2009, 2011, and 2015) of data collection have been conducted.

In this study, we used nine waves of data (from 1991 to 2015) because the 1989 survey only involved children aged <7 and middle-aged adults due to funding constraints. Among 33,453 eligible participants enrolled from 1991 to 2015 survey, we excluded individuals aged <20 y at the entry and those who had null person-years of follow-up, no complete dietary data, or implausible total energy intake (<800 or >4,200 kcal/d for men and <600 or >3,500 kcal/d for women) (13) and those who did not suffer from hypertension at baseline. After exclusion, a total of 8,095 participants were included in the final analysis (Supplementary Figure 1). The study was approved by the institutional review committees of the University of North Carolina and the National Institute of Nutrition and Food Safety, Chinese Center for Disease Control and Prevention. All the participants provided written informed consent.

### Dietary Assessment and Covariates

In each survey, the dietary intakes were measured by the trained-well staff at both the household and individual levels (14, 15). Individual dietary data were collected using 3-d 24-h dietary recalls. All the food consumed at home and away from home, including take-out foods, were weighed and recorded at the beginning and the end of the 3-d 24-h dietary recalls. Cooking oil and spice consumption for each individual was estimated using the weighted household intake. All the nutrient intakes from various foods were assessed and calculated using corresponding versions of the Chinese Food Composition Table (16–18). The cumulative average of a specific food intake was calculated by the sum of intakes from the round when hypertension was first ascertained to the last survey year divided by the number of waves. Intake of a specific nutrient was calculated by the same method. Accumulating averages of the intakes of foods or nutrients could represent the individual long-term dietary patterns and reduce within-person variation.

In view of the potential effect of the dietary pattern on mortality (19), we calculated adjusted Alternative Healthy Eating Index (AHEI), which was composed of nine components: six components for which higher consumption is better [vegetables, fruit, cereal fibers, nuts and legumes, long-chain omega-3 fatty acids, and polyunsaturated fatty acids (PUFAs)]; and three components for which lower consumption is better [sugar-sweetened beverage (SSB), red and processed meat, and sodium) on the basis of Alternative Healthy Eating Index-2010 (AHEI-2010) (20). Each component is given a minimum score of 0 to indicate “worst” intake of that kind(s) of nutrient(s) and is given a maximum score of 10 to indicate “best” intake of that kind(s) of nutrient(s). Therefore, the total score ranged from 0 to 90. Similarly, the cumulative mean of the AHEI score of each available wave was calculated to represent the long-term diet quality. Moreover, a higher AHEI score indicated that the participant had a higher quality of diet.

In a validation study, the correlation coefficient between the total energy intake calculated by the dietary assessment method in the CHNS and that measured by the doubly labeled water method was 0.56 for men and 0.60 for women (both *P* < 0.01) (21). We also divided the dietary cholesterol into cholesterol from eggs and other foods (e.g., red meat, poultry, dairy, nuts/legume, and seafood) to accurately evaluate the effect of various sources of dietary cholesterol.

We also collected other potential health-related factors ranging from the social, demographic to lifestyle factors, and covering age, nationality, sex, weight, height, marital status, annual household income, urbanization index, education degree, alcohol drinking, smoking status, physical activity, and history of hypertension (12). The metabolic equivalent of task hours (MET-h) was calculated using a Compendium of Physical Activities (22) to reflect the physical level. Participants were diagnosed with hypertension if not <1 of the following features occurred: (i) systolic blood pressure above 140 mm Hg; (ii) diastolic blood pressure above 90 mm Hg; (iii) known hypertension; (iv) current hypertensive medication use.

### Death Ascertainment

Death status was ascertained by the report of household members in each survey year. If death was reported repeatedly in different waves of the survey, then the first report was chosen under such situation. Years of follow-up were calculated from the wave at entry until death, the year of censoring, or the end of follow-up (2015), whichever came at first.

### Statistical Analyses

All the nutrients intake was expressed as functions of energy density (g·2,000 kcal^−1^·d^−1^) using the nutrient density method. Egg consumption in grams was converted to numbers of eggs per week (1 egg corresponds to 50 g). Participants were divided into four categories according to the amount of egg consumption. Continuous variables were expressed as mean ± standard error and discrete variables were expressed as percentages of the numbers of participants in each category for egg consumption or quartile for the cholesterol consumption. The chi-squared test (for the discrete variables) and general linear model (for the continuous variables) were used to assess the basic features of the participants according to categories of egg and dietary cholesterol consumption. Time-dependent Cox proportional hazards regression models were utilized to estimate HRs and 95% CIs of mortality in various categories of egg and dietary cholesterol consumption compared with the lowest category. Three stepwise models were used. Model 1 was adjusted for age and sex. Model 2 was further adjusted for the nationality, marital status (never married, married or living as married, widowed/divorced/separated, or unknown), body mass index (BMI, in kg/m^2^; <18.5, 18.5–23.9, 24–27.9, or ≥28), income, urbanization index, education (illiteracy, <high school, or ≥high school), the physical activity (MET-h/wk), smoking status (non-smoker, ever-smoker, and current smoker), alcohol use (yes or no), history of CVD (yes or no), cancer (yes or no), and diabetes (yes or no). Model 3 for the egg consumption was additionally adjusted for the intakes of total energy, cereals, potatoes, dairy products, nuts and legumes, sodium, red meat, white meat, SSB, vegetables, and fruit (all continuous); model 3 for the dietary cholesterol intake was adjusted additionally for the intakes of total energy, proteins, saturated fatty acids (SFAs), PUFAs, monounsaturated fatty acids (MUFAs), sodium and fibers (all continuous). Dietary cholesterol in four quartiles from the eggs and non-eggs was separately analyzed.

Substitution analyses were conducted by theoretically substituting 50 g eggs/d for an equivalent amount of various protein-rich foods, including dairy products, nuts/legumes, red meat, poultry, and seafood. Continuous variables with the total energy intake and other non-diet covariates were included in the substitution analysis. The difference between regression coefficients and in the variances and covariances was used to derive the HRs and 95% CIs of the substitution analyses (23).

Sensitivity analyses were performed to further get rid of the effects of other potential risk factors on the mortality associated with egg and cholesterol intakes among hypertensive patients. The covariates AHEI and anti-hypertensive medication use were further successively considered and adjusted in model 3. Alternatively, we further excluded the participants with extreme BMI (<18.5 or >40 kg/m^2^), with chronic disease (CVD, cancer, or diabetes at baseline), or the deaths during the initial 4 years of follow-up to test whether the results materially changed. Subgroup analyses were then conducted to view whether the associations would vary in the several subgroups stratified by age, sex, BMI, annual household income, physical activity, smoking status, alcohol drinking, or AHEI.

Statistical analyses were performed using the SAS statistical package (version 9.4, SAS Institute). Statistical tests were two-sided and the significance was defined as *P* < 0.05.

## Results

### Population Characteristics

A total of 8,095 participants were included in the final analysis and the average age of the participants was 53.5 ± 13.9 years. Baseline characteristics of the participants across the categories of total egg consumption and the quartiles of total dietary cholesterol intake are shown in Table 1 and Supplementary Table 1, respectively. Participants with higher egg consumption tended to be younger, wealthier, more educated, and reside in more urbanized places; they tended to have a higher BMI and generally consumed higher levels of dairy products, nuts/legumes, red meat, white meat, and fruit (Table 1). Individuals with higher dietary cholesterol consumption appeared to be wealthier, highly educated, and dwelling in a higher degree of urbanized places, and have higher BMI and diabetes, but they were less apt to perform the exercise. In addition, they consumed higher levels of proteins, SFAs, and MUFAs (Supplementary Table 1) compared with those who consumed dietary cholesterol intake with no more than 149 mg·2,000 kcal^−1^·d^−1^.

**Table 1:** Baseline characteristics of the hypertensive patients across categories of egg consumption (*n* = 8,095)^*^.

**Characteristics**	**Categories of egg consumption**				
	**≤2 eggs/wk**	**>2 to ≤4 eggs/wk**	**>4 to ≤7 eggs/wk**	**>7 eggs/wk**	***P*-value**
*n*	2,460	1,630	1,942	2,063	
Age (years)	54.6 ± 0.3	52.9 ± 0.3	52.4 ± 0.3	53.7 ± 0.3	<0.001
Male *n* (%)	1,240 (50.4)	838 (51.4)	1,046 (53.9)	1,136 (55.1)	0.008
Chinese Han^†^ *n* (%)	2,087 (84.8)	1,490 (91.4)	1,799 (92.6)	1,914 (92.8)	<0.001
Body mass index (kg/m^2^)	23.6 ± 0.1	24.2 ± 0.1	24.3 ± 0.1	24.6 ± 0.1	<0.001
Married *n* (%)	1,972 (80.2)	1,381 (84.7)	1,688 (85.9)	1,763 (85.5)	<0.001
≥high school *n* (%)	138 (5.6)	151 (9.3)	210 (10.8)	316 (15.3)	<0.001
Household income (yuan/yr)	27817.5 ± 737.4	33174.9 ± 1199.4	32175.6 ± 853.6	32277.7 ± 804.3	<0.001
Urbanization index	58.5 ± 0.4	63.5 ± 0.5	65.6 ± 0.5	67.6 ± 0.4	<0.001
Physical activity (METs/wk)	170.8 ± 3.6	176.1 ± 4.1	172.9 ± 3.7	163.2 ± 3.5	0.101
Current smoker *n* (%)	820 (33.3)	545 (33.4)	629 (32.4)	668 (32.4)	0.825
Alcohol drinker *n* (%)	890 (36.2)	595 (36.5)	775 (39.9)	847 (41.1)	0.001
Anti-hypertension medicine use *n* (%)	419 (17.0)	316 (19.4)	380 (19.6)	492 (23.9)	<0.001
Cardiovascular disease *n* (%)	95 (3.9)	66 (4.1)	87 (4.5)	99 (4.8)	0.423
Cancer *n* (%)	17 (0.7)	14 (0.9)	9 (0.5)	18 (0.9)	0.401
Diabetes *n* (%)	140 (5.7)	100 (6.1)	140 (7.2)	149 (7.2)	0.096
**Dietary intake**
Total energy (kcal/d)	1971.9 ± 11.6	2036.5 ± 13.5	2059.1 ± 12.0	2098.9 ± 12.2	<0.001
Cereals (g·2,000 kcal^−1^·d^−1^)	519.0 ± 5.0	445.9 ± 3.9	460.7 ± 4.0	475.1 ± 4.6	<0.001
Potatoes (g·2,000 kcal^−1^·d^−1^)	31.4 ± 1.2	32.3 ± 1.3	33.8 ± 1.2	34.5 ± 1.2	0.209
Dairy products (g·2,000 kcal^−1^·d^−1^)	10.9 ± 1.0	16.9 ± 1.4	25.3 ± 1.6	40.5 ± 2.0	<0.001
Nuts/legumes (g·2,000 kcal^−1^·d^−1^)	66.3 ± 1.7	64.6 ± 1.5	67.2 ± 1.6	81.9 ± 1.9	<0.001
Red meat (g·2,000 kcal^−1^·d^−1^)	80.8 ± 1.6	82.2 ± 1.6	83.4 ± 1.7	89.6 ± 1.9	0.001
White meat (g·2,000 kcal^−1^·d^−1^)	48.6 ± 1.6	55.3 ± 1.7	56.9 ± 1.6	56.7 ± 1.6	<0.001
Vegetables (g·2,000 kcal^−1^·d^−1^)	414.7 ± 4.7	372.4 ± 4.8	375.3 ± 4.1	405.7 ± 5.1	<0.001
Fruit (g·2,000 kcal^−1^·d^−1^)	36.7 ± 1.7	48.1 ± 2.0	58.4 ± 2.3	71.7 ± 2.5	<0.001
SSB (g·2,000 kcal^−1^·d^−1^)	6.0 ± 0.6	5.6 ± 0.5	6.0 ± 0.5	6.3 ± 0.5	0.873

* *Data are means ± SE unless otherwise indicated. Household income was inflated to 2015. METs/wk, metabolic equivalent task hours per week; SSB, sugar-sweetened beverage*.

† *Chinese Han is the major ethnic group in China*.

### Egg Consumption and Risk of Mortality

During a total of 92,288 person-years of follow-up, 927 deaths were documented. In an age- and sex-adjusted model (model 1), higher egg intake (> 7 eggs/wk) was associated with a lower risk of mortality (HR: 0.53, 95% CI: 0.45–0.63; *P* < 0.001) compared with the consumers with no more than 2 eggs/wk. After adjustment for the socio-demographic and dietary factors (model 3), such an association appeared to be weaker but still significant (*P* < 0.001). Consuming no <4 eggs/wk was associated with 30 and 29% lower risk of mortality (HR: 0.70, 95% CI 0.58–0.85) for consuming 4–7 eggs/wk and HR: 0.71, 95% CI (0.59–0.85) for consuming more than 7 eggs/wk) compared with the reference category (consuming ≤2 eggs/wk) (Table 2). Similar results were also observed when we divided egg consumption into quartiles (Supplementary Table 2). In detail, participants with a higher egg consumption were inversely related to the incidental death [HR *via* comparing the highest category with the lowest category HR: 0.70 95% CI (0.59–0.84); *P* < 0.001].

**Table 2:** Hazard ratios (HRs) (95% CIs) of the mortality among hypertensive patients according to egg consumption (*n* = 8,095)^*^.

	**Egg consumption**				***P-*trend**
	**≤2 eggs/wk**	**>2 to ≤4 eggs/wk**	**>4 to ≤7 eggs/wk**	**>7 eggs/wk**	
*Case/n*	395/2,460	140/1,630	171/1,942	221/2,063	
Person-years	26,367	18,276	22,429	25,216	
Model 1	1	0.58 (0.47–0.70)	0.58 (0.48–0.69)	0.53 (0.45–0.63)	<0.001
Model 2	1	0.65 (0.53–0.79)	0.69 (0.57–0.83)	0.67 (0.57–0.80)	<0.001
Model 3	1	0.74 (0.61–0.88)	0.70 (0.58–0.85)	0.71 (0.59–0.85)	<0.001

* *Model 1 was adjusted for age and sex*.

*Model 2 was further adjusted for the nationality, marital status (never married, married, or living as married, widowed/divorced/separated, or unknown), BMI, income, urbanization index, education (illiteracy, < high school, or ≥high school), physical activity (MET-h/wk), smoking (non-smoker, ever smoker, and current smoker), alcohol use (yes or no), history of CVD (yes or no), cancer (yes or no), and diabetes (yes or no)*.

*Model 3 was further adjusted for intake of total energy, cereals, potatoes, dairy products, nuts/legumes, sodium, red meat, white meat, SSB, vegetables, and fruit (all continuous)*.

### Dietary Cholesterol and Risk of Mortality

Dietary cholesterol was grouped into cholesterol from eggs and non-eggs (e.g., dairy products, red meat, poultry, nuts/legumes, and seafoods). In the multivariate-adjusted model (model 3), total dietary cholesterol intake was not associated with mortality [HR comparing the highest category (≥413 mg·2,000 kcal^−1^·d^−1^) with the reference category (≤149 mg·2,000 kcal^−1^·d^−1^): 0.94; 95% CI: 0.76–1.16; *P* = 0.92]. However, egg-sourced dietary cholesterol was associated with 44% lower risk of mortality after controlling the effects of age and sex [HR (95% CI) for the highest category (274–5,971 mg·2,000 kcal^−1^·d^−1^) compared with the reference category (≤50 mg·2,000 kcal^−1^·d^−1^): 0.56 (0.48–0.66); *P* < 0.001]. Egg-sourced cholesterol consumption according to the quartiles was also inversely related to the risk of mortality [HR (95% CI): 0.77 (0.64–0.93); *P* = 0.002] in the fully adjusted model (model 3), whereas the risk of death *via* comparing the highest (161–3,382 mg·2,000 kcal^−1^·d^−1^) with the lowest quartile (≤42 mg·2,000 kcal^−1^·d^−1^) was positively associated for the non-egg-sourced cholesterol intake (2.05, 1.58–2.65; *P* < 0.001) (Table 3).

**Table 3:** HRs (95% CIs) of the mortality among hypertensive patients according to the dietary cholesterol intake (*n* = 8,095)^*^.

	**Quartile of dietary cholesterol intake (mg·2,000 kcal^−1^·d^−1^)**				***P-*trend**
	**Q1 (≤149)**	**Q2 (149–264)**	**Q3 (264–413)**	**Q4 (≥413)**	
**Total dietary cholesterol**
Range (mg·2,000 kcal^−1^·d^−1^)	≤ 149	149–264	264–413	413–6,043	
*Cases/n*	304/2,023	189/2,024	202/2,024	232/2,024	
Person-years	22,389	22,618	22,814	24,467	
Model 1	1	0.66 (0.55–0.79)	0.67 (0.56–0.80)	0.54 (0.46–0.64)	<0.001
Model 2	1	0.79 (0.66–0.96)	0.93 (0.77–1.12)	0.78 (0.65–0.94)	0.03
Model 3	1	0.87 (0.72–1.06)	1.06 (0.87–1.29)	0.94 (0.76–1.16)	0.92
**Dietary cholesterol from eggs**
Range (mg·2,000 kcal^−1^·d^−1^)	≤ 50	50–147	147–274	274–5,971	
*Cases/n*	327/2,023	197/2,024	161/2,024	242/2,024	
Person-years	21,131	23,919	23,662	23,576	
Model 1	1	0.62 (0.52–0.74)	0.48 (0.40–0.58)	0.56 (0.48–0.66)	<0.001
Model 2	1	0.66 (0.55–0.79)	0.59 (0.48–0.71)	0.70 (0.59–0.83)	<0.001
Model 3^†^	1	0.69 (0.58–0.83)	0.62 (0.51–0.76)	0.77 (0.64–0.93)	0.002
**Dietary cholesterol from other foods**
Range (mg·2,000 kcal^−1^·d^−1^)	≤ 42	42–94	94–161	161–3,382	
*Cases/n*	289/2,023	206/2,024	196/2,024	236/2,024	
Person-years	23,545	22,081	22,586	24,076	
Model 1	1	0.79 (0.66–0.94)	0.69 (0.57–0.82)	0.72 (0.61–0.86)	<0.001
Model 2	1	0.93 (0.77–1.12)	0.88 (0.73–1.07)	1.15 (0.94–1.39)	0.33
Model 3^‡^	1	1.18 (0.96–1.43)	1.33 (1.06–1.66)	2.05 (1.58–2.65)	<0.001

* *Model 1 was adjusted for the age and sex*.

*Model 2 was further adjusted for nationality, marital status (never married, married or living as married, widowed/divorced/separated, or unknown), BMI, income, urbanization index, education (illiteracy, < high school, or ≥high school), physical activity (MET-h/wk), smoking (non-smoker, ever smoker, and current smoker), alcohol use (yes or no), history of CVD (yes or no), cancer (yes or no), and diabetes (yes or no) at baseline*.

*Model 3 was further adjusted for intake of total energy, proteins, SFAs, MUFAs, PUFAs, sodium, and fibers (all continuous). Q, quartile*.

† *Model 3 was additionally adjusted for the dietary cholesterol from non-egg sources*.

‡ *Model 3 was additionally adjusted for the dietary cholesterol from eggs*.

### Substitution Analyses

**Figure 1:**
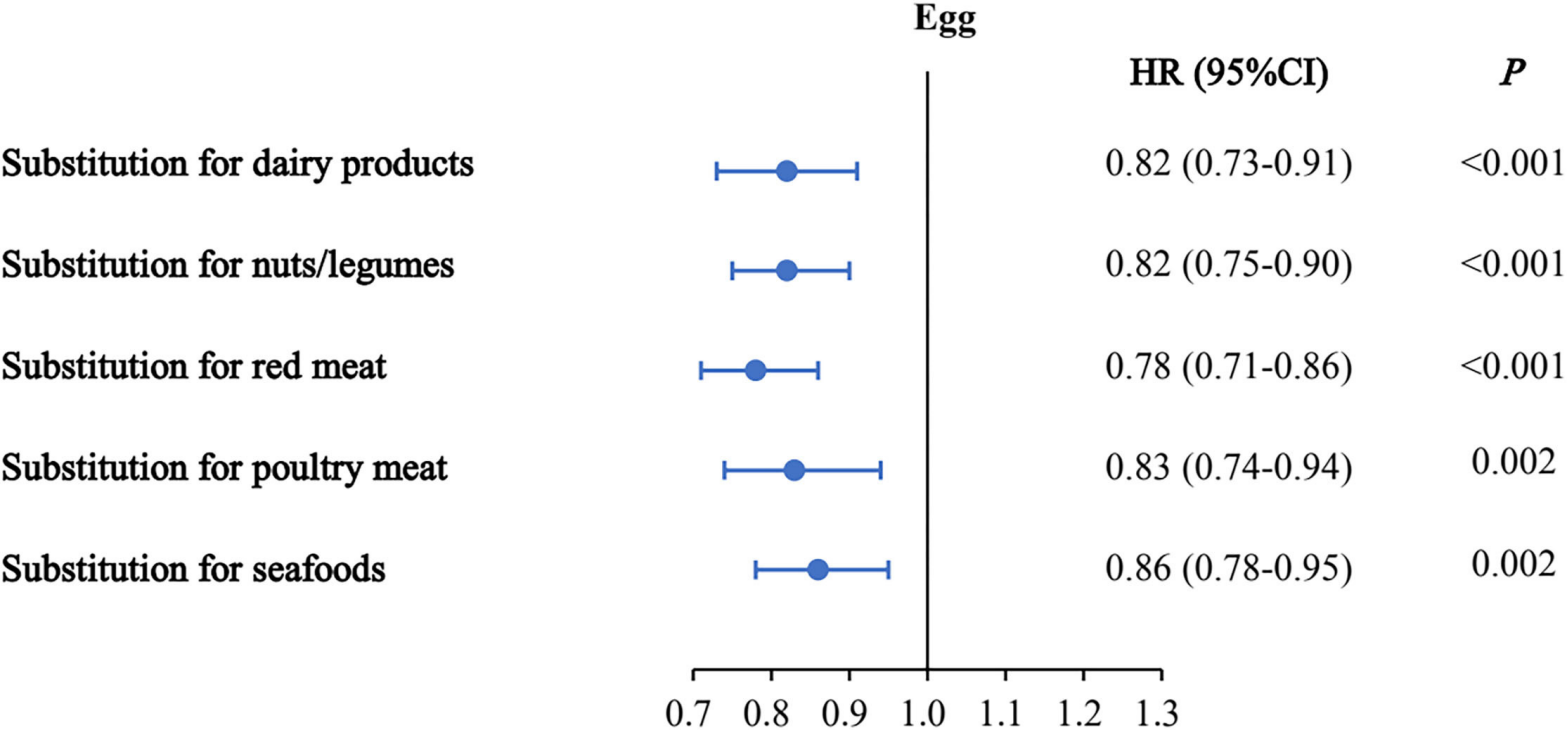
Multivariable hazard ratios (HRs) of mortality by substitution of 50 g/d eggs for the equivalent amount of other protein sources. Forest plots show the multivariable HRs of mortality associated with substituting 50 g/d eggs for equivalent amounts of dairy products, nuts/legumes, red meat, poultry, or seafood. HRs were adjusted for nationality, marital status, body mass index, income, urbanization index, education, smoking, alcohol use, history of cardiovascular disease, cancer, and diabetes, intake of total energy, cereal, potato, dairy product, nut/legume, sodium, red meat, white meat, SSB, vegetables, and fruits at the baseline. Horizontal lines represent 95% CIs.

Hypothetically substituting 50 g/d eggs for the equivalent amounts of dairy products, nuts/legumes, red meat, poultry, and seafoods was related to the reductions of 18, 18, 22, 17, and 14% in the risk of mortality, respectively (Figure 1). Overall, inverse associations between egg consumption and the risk of mortality were still observed after replacing equal amounts of non-egg protein-rich foods with 50 g/d eggs.

### Sensitivity and Subgroup Analyses

Sensitivity analyses that excluded individuals with extreme BMIs, chronic diseases at baseline, or the deaths within the initial 4 years during the follow-up and that further adjusted for the dietary pattern assessed by AHEI and current hypertensive medication use validated the robustness of our findings. The results were not materially changed (Supplementary Table 3). Subgroup analyses that were stratified by age, sex, BMI, physical activity, household income, smoking status, alcohol drinking, and AHEI were also performed. Regarding the relationship between egg consumption and mortality, we did not find significant differences among various subgroups (all *P* > 0.05) (Supplementary Table 4).

## Discussion

To our knowledge, the current 14-year prospective study is the first report to longitudinally assess the associations of egg and dietary cholesterol intakes with mortality among hypertensive patients in a Chinese population. We found that a higher intake of an egg was related to the lower risk of mortality among the participants with hypertension. Total dietary cholesterol intake was not associated with the risk of mortality. Surprisingly, the findings of the associations of egg-sourced and non-egg-sourced cholesterol intakes with mortality among the hypertensive patients appeared completely opposite.

Previous studies reporting the association between egg consumption and mortality were inconsistent. During the mean follow-up duration of 22.8 years in the Caerphilly Prospective Cohort Study (CAPS), 1,028 cases of death occurred and no association between weekly egg consumption (1–2 eggs/wk up to 5 eggs/wk) and risk of total mortality was observed (24). Similarly, the Spanish European Prospective into Cancer and Nutrition (EPIC-Spain) study indicated that the egg consumption was not associated with all-cause, cancer, and CVD mortality *via* comparing the highest (>42.6 g/d for men and >30.4 g/d for women) with the lowest quartile (<14.4 g/d for men and <10.8 g/d for women) (*P* = 0.96) (25). Nonetheless, in a prospective cohort study consisting of 21,327 participants from Health Study of the Physicians, the author showed that higher egg consumption was positively related to mortality when comparing those who consumed at least 7 eggs/wk with the consumers with <1 egg/wk (*P* < 0.001), and this association was stronger in the diabetic patients (7). The discrepancy may be attributable to confounding variables or differences in the dietary patterns between populations. For example, the Mediterranean population develops the Mediterranean diet featuring a high consumption of whole cereals, legumes and nuts, vegetables, fruit, moderate consumption of dairy products, and red wine, and low consumption of red meat and poultry (26). Massive studies advocated benefits of the adherence to the Mediterranean diet on human health-related outcomes, including total mortality, CHD mortality, and the risk of CVD, type 2 diabetes, hepatic steatosis, or bladder cancer (27–31). However, the Western diet was popular among Americans and other non-Western countries and featured high consumption of sodium, sugar, and saturated fat (32). This kind of suboptimal diet was associated with a higher risk of obesity and hepatic steatosis on the basis of findings from animal studies (33, 34). Recent summary analysis from 14 studies showed that intake of up to 6 eggs/wk was inversely associated with CVD risk (incidence and/or mortality) (9), while we still found a significant protective association between >7 eggs/wk intake and the risk of death. Besides, the role of egg intake in health outcomes was also reported among the Asian population. On the contrary, a meta-analysis study that utilized data from the China Kadoorie Biobank study of 0.5 million Chinese adults showed that compared with the non-consumers, daily egg consumers (usually 0.76 egg/d) had reductions of 18, 28, 10, and 12% in the risks of CVD death, hemorrhagic stroke death, ischemic stroke, and ischemic heart disease (IHD), respectively (35). Researchers found that consuming 1 egg/d was not associated with a higher risk of CVD, subtypes of CVD (stroke and IHD), or all-cause mortality, and also a small reduction in stroke was observed in the Guangzhou Biobank Cohort Study in China (36). Likewise, eating eggs more frequently, up to almost daily, was not related to an increase in CHD incidence for the middle-aged Japanese according to results from the Japan Public Health Center-based prospective study (37). These findings indicated that egg consumption was not associated with a higher incidence of cardio- and cerebrovascular diseases among the Asian population, which was in agreement with our main results that higher egg consumption had a reduction in the risk of mortality among the Chinese hypertensive patients.

Egg intake is debatable mainly because of its high content of cholesterol. Given results from a series of studies showed that higher total cholesterol (TC) intake was related to the elevated risk of cardio- and cerebrovascular diseases or total mortality (10, 38, 39), people are concerned about an excess of cholesterol consumption along with the egg intake. However, a population-based cohort study (Swedish National Study on Aging and Care in Kungsholmen, Stockholm) revealed that high TC level (≥6.22 mmol/l) was inversely related to the all-cause mortality, primarily due to non-cardiovascular mortality (37), while no association existed between the high TC levels ≥60 mg/dl and liver cancer-related mortality from a large pooled analysis of 12 cohorts in Japan (40). In addition, the epidemiological studies showed that the dietary cholesterol was not associated with CHD incidence or mortality across or within populations primarily due to the non-significant effect of dietary cholesterol on the ratio of low-density lipoprotein (LDL) to high-density lipoprotein (HDL), a major determinant of the heart disease risk. Given the limited evidence supporting the causal effect of dietary cholesterol on CVD risk, the 2015 Dietary Guidelines Advisory Committee Report and the Chinese Dietary Guidelines (2016) lifted the restriction on maximum intake of 300 mg/d of the dietary cholesterol (41). Here, we found that TC consumption was also not associated with mortality among individuals with hypertension, which is in line with the previous studies. Notably, most dairy and meat products including red meat, poultry, cheese, and butter with the exception of eggs and shrimp that are abundant with cholesterol are also high in SFAs, and lines of evidence from the epidemic studies revealed that SFA or its subtypes, including palmitic acids and myristic acids may increase the risk of CVD or mortality (42–45).

In the clinical intervention trials, researchers found that cholesterol from eggs could maintain the ratio of LDL to HDL and thus regulated endogenous synthesis of cholesterol *via* comparing the subjects with 3 eggs/d with those who consumed choline bitartrate supplement (46) or comparing the subjects with 2 eggs/d with those who ate an oatmeal breakfast (47). In addition, two randomized-crossover studies separately suggested consuming cooked whole eggs did not affect the areas under the curve (AUC_0−10h_) of plasma TC, indicating that cholesterol from whole eggs was not well-absorbed (48). A similar result that high egg consumption (2 eggs/d for 6 d/wk) had no adverse effect on the lipid profile was observed among the patients with type 2 diabetes (49). Furthermore, the results from a hamster experiment investigated the dose-dependent effect of consuming 0–5 whole eggs on plasma TC found that the consumption of 2–5 eggs did not significantly affect plasma TC levels (50). Diverse populations had different responses to the dietary cholesterol and only 25% population (hyper-responders) experienced an increase in both LDL-C and HDL-C (51). The rest of the population had a mild increase or no alterations in the plasma cholesterol concentrations when consumed dietary cholesterol. This result supported our finding of the overall non-significant relationship between TC intake and mortality.

In addition, the egg is not only low in SFAs but also nutrient-dense. A large whole egg (50 g) generally contains 1.56 g of SFAs, 1.83 g of MUFAs, and 0.96 g of PUFAs (42). The concentration of dietary choline in the yolk is also abundant, while the higher dietary choline intake was reported to be associated with the lower ischemic stroke incidence, and promote neurological health and brain development of the children (52–54). Besides, eggs are abundant in other beneficial components including vitamins (A, B, and D), carotenoids, and minerals, which could have contributed to longevity. A meta-analysis of the randomized controlled trials revealed a beneficial effect of vitamin D supplementation on the risk of cancer [RR (95% CI): 0.84 (0.74–0.95), *I*^2^ = 0%] (55). In addition, the egg components such as carotenoids, lutein, and zeaxanthin played a significant role in the anti-inflammatory process (5). It may be due to these beneficial components that the association of egg-sourced cholesterol intake with the risk of death was significantly inverse. These findings may also explain the reason why the associations of egg-sourced and non-egg-sourced dietary cholesterol intakes with mortality among the hypertensive patients obviously varied.

The strengths of this study included the prospective design, a high participation rate of up to 88%, a long duration of follow-up, and detailed information on potential confounders. Our current study also calculated the cumulative average of egg and cholesterol consumption during all the available waves to minimize the influence of changes in dietary habits. This study did have several limitations. First, we cannot further analyze the associations between egg and cholesterol intakes and cause specific (e.g., CVD, CHD, and stroke) mortality because of the lack of data. Second, the reverse causation may bias our findings. Nonetheless, we further excluded participants with CVD, cancer, or diabetes at baseline, or deaths that occurred during the initial 4 years of follow-up in the sensitivity analyses and found no significant changes. Third, although we adjusted for the dietary pattern represented by AHEI, we still could not cover the effects of the dietary pattern and interactions between various nutrients. For example, the Chinese prefer consuming eggs with pure milk or soybean, and although consumption of the dairy and soybean products were considered as covariates, the interactions still cannot be fully eliminated. Fourth, our findings may not apply to other hypertensive populations because of the different long-term dietary habits in various countries. Fifth, the measurement errors were still inevitable that may dilute the real association between eggs intake and risk of mortality to the null. Finally, a causal association cannot be proved in our study due to the observational nature, and residual confounding is still possible even though we have fully controlled most of the potential risk factors.

In summary, we identified the protective role of egg consumption in mortality among hypertensive patients in the Chinese population. People should not be too concerned about cholesterol consumption from egg yolks; at least, due to eggs as an economical and nutrient-rich food, consuming one egg per day is appropriate and beneficial for persons with hypertension. However, imposing a limit on the dietary cholesterol consumption from non-eggs such as red meat should keep forward. Our findings provide convincing evidence for the amendment of the Chinese dietary guidelines in the future.

## Data Availability Statement

The raw data supporting the conclusions of this article will be made available by the authors, without undue reservation.

## Ethics Statement

The studies involving human participants were reviewed and approved by University of North Carolina and the National Institute of Nutrition and Food Safety and Chinese Center for Disease Control and Prevention. The patients/participants provided their written informed consent to participate in this study.

## Author Contributions

FW and PZ performed the analysis. FW wrote the manuscript. PZ, YJZ, and CCZ contributed to the data analysis. YZ and JJJ designed the study and critically revised the manuscript. YZ is the guarantor of this work and, as such, has full access to all the data in the study and takes responsibility for the integrity of the data and the accuracy of the data analysis. All authors read and approved the manuscript.

## Funding

This research was funded by grants from the Zhejiang Provincial National Natural Science Foundation of China (Grant No. LR18C200001). We are also grateful to the research grant funding from the National Institute for Health (NIH), the Eunice Kennedy Shriver National Institute of Child Health and Human Development (NICHD, R01 HD30880; P2C HD050924), the National Institute of Diabetes and Digestive and Kidney Diseases (NIDDK, R01 DK104371), the NIH Fogarty D43 TW009077 for financial support for the CHNS data collection and analysis files since 1989, the China-Japan Friendship Hospital, Ministry of Health for support for CHNS 2009, Chinese National Human Genome Center at Shanghai since 2009, and Beijing Municipal Center for Disease Prevention and Control since 2011. We thank the National Institute for Nutrition and Health, China Center for Disease Control and Prevention.

## Conflict of Interest

The authors declare that the research was conducted in the absence of any commercial or financial relationships that could be construed as a potential conflict of interest.

## Publisher's Note

All claims expressed in this article are solely those of the authors and do not necessarily represent those of their affiliated organizations, or those of the publisher, the editors and the reviewers. Any product that may be evaluated in this article, or claim that may be made by its manufacturer, is not guaranteed or endorsed by the publisher.
