# Supplementary material for: Egg and Dietary Cholesterol Consumption and Mortality Among Hypertensive Patients: Results From a Population-Based Nationwide Study
Source: Front Nutr. 2021 Oct 29;8:739533. doi: 10.3389/fnut.2021.739533 (PMC8588794; doi:10.3389/fnut.2021.739533)
Supplement: Supplementary file 1 [file Data_Sheet_1.docx]

**Supplementary Material**

**Egg and dietary cholesterol consumption and mortality among hypertensive patients in China: Results from a population-based nationwide study**

Fei Wu, Pan Zhuang, Yiju Zhang, Chuchu Zhan, Yu Zhang, Jingjing Jiao*

**Table of Contents**

**Supplementary Figure 1.** Flow chart of study participants in CHNS.

**Supplementary Table 1.** Baseline characteristics of hypertensive patients across the quartiles of dietary cholesterol intake (*n*=8095).

**Supplementary Table 2.** HRs (95% CIs) of mortality among hypertensive patients across the quartiles of egg consumption (*n*=8095).

**Supplementary Table 3.** Sensitivity analyses for the associations between egg consumption and mortality among hypertensive patients.

**Supplementary Table 4.** Subgroup analyses for the associations between egg consumption and mortality among hypertensive patients.

**Supplementary Figure 1.** Flow chart of study participants in CHNS.


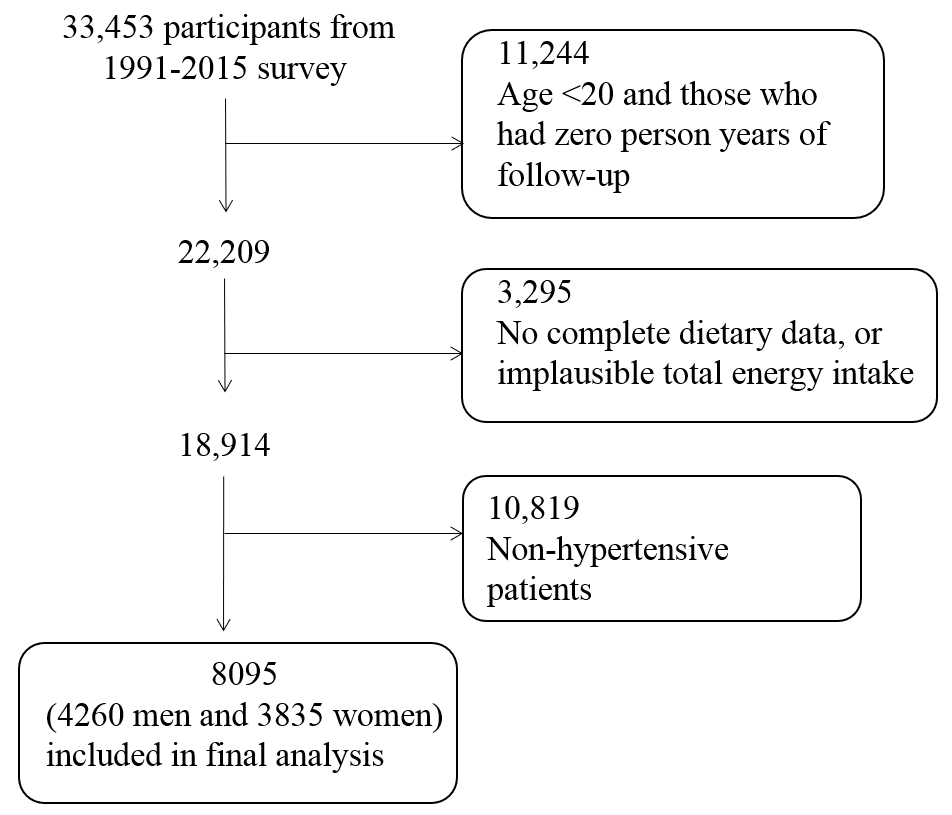


**Supplementary Table 1.** Baseline characteristics of hypertensive patients across the quartiles of dietary cholesterol intake (*n*=8095)*

| **Characteristics** | **Quartiles of dietary cholesterol intake (mg·2000 kcal^-1^·d^-1^)** | | | | |
| --- | --- | --- | --- | --- | --- |
|  | **Q1 (≤149)** | **Q2 (149-264)** | **Q3 (264-413)** | **Q4 (≥413)** | **P value** |
| *n* | 2023 | 2024 | 2024 | 2024 |  |
| Age (years) | 53.7±0.3 | 51.8±0.3 | 52.8±0.3 | 55.6±0.3 | <0.001 |
| Male n (%) | 1028(50.8) | 1126(55.6) | 1103(54.5) | 1003(49.6) | <0.001 |
| Chinese Han n (%) | 1728(85.4) | 1819(89.9) | 1867(92.2) | 1876(92.7) | <0.001 |
| Body mass index (kg/m^2^) | 23.5±0.1 | 24.0±0.1 | 24.6±0.1 | 24.5±0.1 | <0.001 |
| Married n (%) | 1617(79.9) | 1736(85.8) | 1749(86.4) | 1682(83.1) | <0.001 |
| ≥high school n (%) | 78(3.9) | 166(8.2) | 251(12.4) | 320(15.8) | <0.001 |
| Household income (yuan/yr) | 22773.8±824.4 | 30380.2±789.8 | 34669.6±918.7 | 36515.6±943.1 | <0.001 |
| Urbanization index | 52.1±0.5 | 62.6±0.5 | 68.5±0.4 | 70.9±0.4 | <0.001 |
| Physical activity (METs/wk) | 184.1±4.1 | 193.0±3.9 | 163.1±3.3 | 141.5±3.3 | <0.001 |
| Current smoker n (%) | 668(34.0) | 716(35.4) | 656(32.4) | (602)29.7 | <0.001 |
| Alcohol drinker n (%) | 713(35.2) | 846(41.8) | 794(39.2) | (754)37.3 | 0.001 |
| anti-hypertensive medication use n (%) | (258)12.8 | (358)17.7 | (434)21.4 | (557)27.5 | <0.001 |
| Cardiovascular disease n (%) | (77)3.8 | (73)3.6 | (99)4.9 | (98)4.8 | 0.074 |
| Cancer n (%) | (13)0.6 | (8)0.4 | (18)0.9 | (19)0.9 | 0.692 |
| Diabetes n (%) | (111)5.5 | (107)5.3 | (147)7.3 | (164)8.1 | 0.001 |
| **Dietary intake** |  |  |  |  |  |
| Total energy (kcal/day) | 2081.7±13.5 | 2127.9±12.3 | 2038.8±11.0 | 1904.3±11.9 | <0.001 |
| Proteins (% of energy) | 11.3±0.0 | 12.3±0.1 | 13.0±0.1 | 14.5±0.1 | <0.001 |
| SFAs (% of energy) | 6.8±0.1 | 8.6±0.1 | 9.5±0.1 | 10.0±0.1 | <0.001 |
| PUFAs (% of energy) | 8.3±0.1 | 9.2±0.1 | 9.5±0.1 | 9.5±0.1 | 0.060 |
| MUFAs (% of energy) | 10.9±0.1 | 13.1±0.1 | 14.2±0.1 | 14.5±0.1 | <0.001 |
| Sodium (g·2000 kcal^-1^·d^-1^) | 5210.7±432.3 | 4644.1±181.2 | 5579.9±788.6 | 5665.4±287.8 | 0.409 |
| Fibers (g·2000 kcal^-1^·d^-1^) | 13.6±0.2 | 12.2±0.2 | 12.1±0.1 | 12.7±0.2 | 0.211 |
| Cholesterol from eggs  (mg·2000 kcal^-1^·d^-1^) | 30.0±0.9 | 108.8±1.5 | 201.0±2.0 | 482.0±8.3 | <0.001 |
| Cholesterol from other foods (mg·2000 kcal^-1^·d^-1^) | 46.3±0.9 | 97.7±1.4 | 131.6±1.8 | 191.3±4.0 | 0.147 |

* Data are means ± SE unless otherwise indicated. Household income was inflated to 2015. METs/wk, metabolic equivalent task hours per week; SSB, sugar-sweetened beverage.

**Supplementary Table 2.** HRs (95% CIs) of mortality among hypertensive patients across the quartiles of egg consumption (*n*=8095)*

|  | **Egg consumption (g·2000 kcal^-1^·d^-1^)** | | | | ***P*-trend** |
| --- | --- | --- | --- | --- | --- |
|  | **Q1** | **Q2** | **Q3** | **Q4** |  |
| Range (g·2000 kcal^-1^·d^-1^) | ≤9.5 | 9.5-27.5 | 27.5-52.1 | ≥52.2 |  |
| *Cases/n* | 326/2023 | 196/2024 | 165/2024 | 240/2024 |  |
| Person-years | 21167 | 23839 | 23567 | 23715 |  |
| Model 1 | 1.00 | 0.61 (0.51-0.73) | 0.50 (0.41-0.60) | 0.55 (0.46-0.65) | <0.001 |
| Model 2 | 1.00 | 0.66 (0.55-0.79) | 0.62 (0.51-0.76) | 0.69 (0.58-0.82) | <0.001 |
| Model 3 | 1.00 | 0.71 (0.59-0.87) | 0.75 (0.63-0.91) | 0.70 (0.59-0.84) | <0.001 |

* Model 1 was adjusted for age and sex. Model 2 was further adjusted for nationality, marital status (never married, married or living as married, widowed/divorced/separated, or unknown), BMI, income, urbanization index, education (illiteracy, <high school, or ≥high school), physical activity (MET-h/wk), smoking (non-smoker, ever-smoker, current smoker), alcohol use (yes or no), history of CVD (yes or no), cancer (yes or no) and diabetes (yes or no). Model 3 was further adjusted for intake of total energy, cereals, potatoes, dairy products, nuts/legumes, sodium, red meat, white meat, SSB, vegetables, and fruit (all continuous). CI, confidence interval; HR, hazard ratio; Q, quartile.

**Supplementary Table 3****.** Sensitivity analyses for the associations between egg consumption and mortality among hypertensive patients*

|  | **Egg consumption** | | | | ***P-*trend** |
| --- | --- | --- | --- | --- | --- |
|  | **Q1** | **Q2** | **Q3** | **Q4** |  |
| **Further adjusted for AHEI (minus component for alcohol intake)** | | | | | |
| Eggs | 1.00 | 0.66 (0.56-0.80) | 0.62 (0.51-0.75) | 0.66 (0.55-0.79) | <0.001 |
| Total cholesterol | 1.00 | 0.80 (0.67-0.96) | 0.93 (0.77-1.12) | 0.76 (0.63-0.92) | 0.02 |
| Cholesterol from eggs | 1.00 | 0.66 (0.55-0.79) | 0.58 (0.48-0.70) | 0.67 (0.56-0.80) | <0.001 |
| Cholesterol from other foods | 1.00 | 0.95 (0.78-1.14) | 0.91 (0.75-1.11) | 1.21 (0.99-1.48) | 0.14 |
| **Further adjusted for anti-hypertensive medication use** | | | | | |
| Eggs | 1.00 | 0.74 (0.61-0.88) | 0.70 (0.58-0.85) | 0.71 (0.59-0.85) | <0.001 |
| Total cholesterol | 1.00 | 0.87 (0.72-1.06) | 1.06 (0.87-1.29) | 0.94 (0.76-1.16) | 0.92 |
| Cholesterol from eggs | 1.00 | 0.69 (0.58-0.83) | 0.62 (0.51-0.76) | 0.80 (0.66-0.96) | 0.004 |
| Cholesterol from other foods | 1.00 | 1.19 (0.98-1.45) | 1.34 (1.08-1.67) | 2.05 (1.59-2.64) | <0.001 |
| **Excluding extreme BMIs** | | | | | |
| Eggs | 1.00 | 0.69 (0.56-0.84) | 0.67 (0.54-0.82) | 0.70 (0.58-0.85) | <0.001 |
| Total cholesterol | 1.00 | 0.79 (0.64-0.97) | 1.04 (0.84-1.28) | 0.91 (0.72-1.14) | 0.85 |
| Cholesterol from eggs | 1.00 | 0.68 (0.56-0.83) | 0.59 (0.47-0.73) | 0.82 (0.67-1.01) | 0.01 |
| Cholesterol from other foods | 1.00 | 1.21 (0.98-1.50) | 1.30 (1.02-1.65) | 2.00 (1.52-2.64) | <0.001 |
| **Excluding chronic diseases** **at baseline ^2^** | | | | | |
| Eggs | 1.00 | 0.78 (0.65-0.95) | 0.69 (0.56-0.85) | 0.71 (0.59-0.86) | <0.001 |
| Total cholesterol | 1.00 | 0.87 (0.71-1.07) | 1.03 (0.84-1.28) | 0.94 (0.75-1.19) | 0.94 |
| Cholesterol from eggs | 1.00 | 0.69 (0.56-0.83) | 0.63 (0.52-0.78) | 0.81 (0.66-0.98) | 0.01 |
| Cholesterol from other foods | 1.00 | 1.24 (1.00-1.53) | 1.46 (1.16-1.85) | 2.33 (1.78-3.06) | <0.001 |
| **Excluding deaths within the initial 4 years** | | | | | |
| Eggs | 1.00 | 0.80 (0.66-0.98) | 0.78 (0.63-0.96) | 0.75 (0.62-0.92) | 0.005 |
| Total cholesterol | 1.00 | 0.88 (0.71-1.09) | 1.15 (0.93-1.43) | 0.94 (0.74-1.19) | 0.86 |
| Cholesterol from eggs | 1.00 | 0.76 (0.63-0.93) | 0.70 (0.57-0.87) | 0.85 (0.69-1.05) | 0.07 |
| Cholesterol from other foods | 1.00 | 1.24 (1.00-1.55) | 1.34 (1.05-1.71) | 2.03 (1.54-2.70) | <0.001 |

* The multivariate-adjusted model was adjusted for age, sex, nationality, marital status (never married, married or living as married, widowed/divorced/separated, or unknown), BMI, income, urbanization index, education (illiteracy, <high school, or ≥high school), physical activity (MET-h/wk), smoking (non-smoker, ever-smoker, current smoker), alcohol use (yes or no), history of CVD (yes or no), cancer (yes or no) and diabetes, intake of total energy, cereals, potatoes, dairy products, nuts/legumes, sodium, red meat, white meat, SSB, vegetables, and fruit (all continuous). AHEI, Alternative Healthy Eating Index; BMI, body mass index.

^2^ Chronic disease including cardiovascular disease, cancers and diabetes.

**Supplementary Table 4.** Subgroup analyses for the associations between egg consumption and mortality among hypertensive patients*

| **Subgroups** | **Egg consumption** | | | | ***P-*trend** | ***P*-interaction** |
| --- | --- | --- | --- | --- | --- | --- |
|  | **Q1** | **Q2** | **Q3** | **Q4** |  |  |
| **Age** |  |  |  |  |  | 0.49 |
| <60 years | 1.00 | 0.72 (0.52-1.00) | 0.82 (0.58-1.16) | 0.98 (0.70-1.37) | 0.89 |  |
| ≥60 years | 1.00 | 0.80 (0.64-1.00) | 0.66 (0.52-0.84) | 0.66 (0.53-0.81) | <0.001 |  |
| **Sex** |  |  |  |  |  | 0.94 |
| Men | 1.00 | 0.72 (0.56-0.93) | 0.73 (0.56-0.94) | 0.73 (0.57-0.93) | 0.01 |  |
| Women | 1.00 | 0.71 (0.54-0.94) | 0.66 (0.48-0.89) | 0.66 (0.50-0.86) | 0.002 |  |
| **BMI** |  |  |  |  |  | 0.93 |
| <24 | 1.00 | 0.72 (0.57-0.89) | 0.69 (0.54-0.88) | 0.72 (0.58-0.90) | 0.002 |  |
| ≥24 | 1.00 | 0.73 (0.52-1.04) | 0.71 (0.50-1.00) | 0.66 (0.47-0.91) | 0.02 |  |
| **Household income** |  |  |  |  |  | 0.92 |
| <Median | 1.00 | 0.70 (0.56-0.87) | 0.70 (0.55-0.88) | 0.69 (0.56-0.85) | <0.001 |  |
| ≥Median | 1.00 | 0.85 (0.60-1.20) | 0.72 (0.51-1.04) | 0.74 (0.53-1.03) | 0.06 |  |
| **Physical activity** |  |  |  |  |  | 0.11 |
| <Median | 1.00 | 0.70 (0.57-0.87) | 0.68 (0.55-0.86) | 0.66 (0.54-0.80) | <0.001 |  |
| ≥Median | 1.00 | 0.80 (0.55-1.17) | 0.72 (0.48-1.08) | 1.03 (0.68-1.56) | 0.77 |  |
| **Smoking** |  |  |  |  |  | 0.29 |
| Non/former smoker | 1.00 | 0.81 (0.65-1.02) | 0.64 (0.50-0.83) | 0.68 (0.55-0.86) | <0.001 |  |
| Current smoker | 1.00 | 0.62 (0.45-0.84) | 0.75 (0.55-1.03) | 0.76 (0.56-1.03) | 0.14 |  |
| **Alcohol drinking** |  |  |  |  |  | 0.77 |
| Nondrinker | 1.00 | 0.71 (0.56-0.90) | 0.71 (0.56-0.91) | 0.68 (0.54-0.85) | <0.001 |  |
| Drinker | 1.00 | 0.79 (0.59-1.07) | 0.70 (0.50-0.97) | 0.79 (0.58-1.07) | 0.08 |  |
| **AHEI** |  |  |  |  |  | 0.71 |
| <Median | 1.00 | 0.86 (0.66-1.12) | 0.77 (0.58-1.04) | 0.79 (0.60-1.04) | 0.06 |  |
| ≥Median | 1.00 | 0.62 (0.48-0.80) | 0.61 (0.47-0.80) | 0.60 (0.47-0.76) | <0.001 |  |

* The multivariate-adjusted model was adjusted for age, sex, nationality, marital status (never married, married or living as married, widowed/divorced/separated, or unknown), BMI, income, urbanization index, education (illiteracy, <high school, or ≥high school), physical activity (MET-h/wk), smoking (non-smoker, ever-smoker, current smoker), alcohol use (yes or no), history of CVD (yes or no), cancer (yes or no) and diabetes, intake of total energy, cereals, potatoes, dairy products, nuts/legumes, sodium, red meat, white meat, SSB, vegetables, and fruit (all continuous). AHEI, Alternative Healthy Eating Index; BMI, body mass index.
